# Time-Preference Tests Fail to Predict Behavior Related to Self-control

**DOI:** 10.3389/fpsyg.2017.00150

**Published:** 2017-02-09

**Authors:** Kodi B. Arfer, Christian C. Luhmann

**Affiliations:** ^1^UCLA Center for HIV Identification, Prevention, and Treatment Services, University of California, Los AngelesLos Angeles, CA, USA; ^2^Psychology Department, Stony Brook UniversityStony Brook, NY, USA

**Keywords:** decision making, intertemporal choice, self-control, retest reliability, predictive validity

## Abstract

According to theory, choices relating to patience and self-control in domains as varied as drug use and retirement saving are driven by generalized preferences about delayed rewards. Past research has shown that measurements of these time preferences are associated with these choices. Research has also attempted to examine how well such measurements can predict choices, but only with inappropriate analytical methods. Moreover, it is not clear which of the many kinds of time-preference tests that have been proposed are most useful for prediction, and a theoretically important aspect of time preferences, nonstationarity, has been neglected in measurement. In Study 1, we examined three approaches to measuring time preferences with 181 users of Mechanical Turk. Retest reliability, for both immediate and 1-month intervals, was decent, as was convergent validity between tests, and association was similar to previous results, but predictive accuracy for 10 criterion variables (e.g., tobacco use) was approximately nil. In Study 2, we examined one other approach to measuring time preferences, and 40 criterion variables, using 7,127 participants in the National Longitudinal Survey of Youth 1979. Time preferences were significantly related to criterion variables, but predictive accuracy was again poor. Our findings imply serious problems for using time-preference tests to predict real-world decisions. The results of Study 1 further suggest there is little value in measuring nonstationarity separately from patience.

## 1. Introduction

### 1.1. Measuring time preferences

People frequently need to choose between one outcome available soon (the smaller sooner, or SS, option) and a more desirable outcome available later (the larger later, or LL, option). Deciding whether to indulge in a dessert or stick to a diet, to splurge on an impulse buy or save up for a more desirable item, and to relax or study for an upcoming exam, can all be characterized as intertemporal choices. Intertemporal choice is often called “delay discounting,” because of people's and animals' tendency to treat LL as less valuable the more it is delayed, which has led to the development and popularization of quantitative models of intertemporal choice based on multiplicative discount factors (Samuelson, 1937; Mazur, 1987; Rachlin et al., 1991). A long-influential idea in the study of decision-making is that people have stable individual differences in how they make intertemporal choices (Myerson and Green, 1995). In particular, it is theorized that such *time preferences* are a key determinant of success in self-control dilemmas, such as attempting to cease use of an addictive drug, because in such cases the individual must choose between the SS reward of succumbing to temptation and the LL reward of keeping good habits (Kirby et al., 1999). This thinking is supported by findings that measurements of time preferences are associated in expected directions with variables such as body mass index (Sutter et al., 2010), credit-card debt (Meier and Sprenger, 2010), and heroin addiction (Madden et al., 1997).

It is generally agreed that a high-quality standardized test of a personality construct is important for research into that construct. Actually, while time preferences have been studied from such diverse perspectives as cognitive psychology, economics, and psychiatry, they have not generally been regarded as a personality construct (see Odum, 2011 for an exception). And yet, time preferences have a rich underlying theory, they are associated with many other variables, and individual differences are stable over time, seemingly qualifying them as personality constructs par excellence. Evidence of stability over time comes from studies of retest reliability. Kirby (2009) found that administrations separated by a year produced logged discount rates correlated 0.71. Beck and Triplett (2009) found a correlation of 0.64 after a 6-week interval. Meier and Sprenger (2015) found that, collapsing across subjects, subjects made the same choice in 73% of pairs of identical trials offered a year apart.

Despite widespread interest in time preferences and use of tests to measure them, there has been little agreement on which tests to use. Instead, researchers have employed a wide variety of tests. Some tests have subjects make binary choices (Rachlin and Green, 1972; Kirby et al., 1999), whereas others have subjects write in values that would make them indifferent between two options (Kirby and Marakovic, 1995; Kimura et al., 2013). Some tests consider only SS delays of 0, whereas other tests consider scenarios in which SS as well as LL is delayed (Rachlin and Green, 1972; McClure et al., 2004). Some tests give all subjects the same items, whereas others use a variety of strategies to choose items adaptively, hopefully yielding more reliable measurements with fewer trials (Logan, 1965; Toubia et al., 2013; Koffarnus and Bickel, 2014; Lu et al., 2014). Some tests have subjects make decisions about hypothetical events, whereas others give subjects real payments after real delays, whether those delays are on the scale of days (Kirby and Marakovic, 1995; Read et al., 2012) or of seconds between trials of a multiple-trial task (Rachlin and Green, 1972; Reynolds and Schiffbauer, 2004; Johnson, 2012). Finally, scoring procedures have varied from simple consistency measures to maximum likelihood estimation (cf. Kirby, 2009; Myerson et al., 2001; Zauberman et al., 2009).

Given this wide array of possibilities, what test of time preferences should an investigator use? Past research has examined various aspects of how time preferences are measured (such as forced-choice vs. free-response formats), how these measurement details affect the resulting scores, and how well scores obtained from different tests agree (e.g., Smith and Hantula, 2008; Weatherly and Derenne, 2011; Weatherly, 2014). To our knowledge, however, no past work has examined how such measurement details affect reliability or criterion validity. Reliability and criterion validity are key determinants of how useful a test will be in practice, and therefore should guide our choice of test.

One way in which the many extant tests are similar, but possibly deficient, is that all but a few of them measure only patience, that is, people's overall willingness to wait for rewards. Patience is distinct from nonstationarity, which is how people's patience changes as the delays to both SS and LL increase in equal measure. It is nonstationarity that is theoretically linked to self-control (Thaler and Shefrin, 1981; Ariely and Wertenbroch, 2002); a person who is impatient but stationary, such as a person who always says they prefer watching TV to exercising (rather than first saying they prefer exercising and then changing their mind when the time comes), is not thought of as lacking self-control, but as generally preferring immediate gratification. A self-control problem, then, is when people change their minds from patient to impatient choices as a scenario draws closer. Accommodation of nonstationarity is an important strength of the hyperbolic delay-discounting model that is influential in research on economic decision-making (Ainslie, 1975; Mazur, 1987; Ainslie, 2001). If the economic theory of time preferences' effect on self-control is correct, measuring nonstationarity may greatly increase a time-preference test's criterion validity for behavior related to self-control.

### 1.2. Association vs. prediction

A statistical issue that is particularly important for criterion validity and applied uses of tests is the distinction between association and predictive accuracy. Typically, when psychologists wish to quantify the relationship between variables, they use a measure of how well values can be associated with each other, such as a correlation coefficient, or how well a model fits the data, such as root mean squared error (RMSE). The question of predictive accuracy, by contrast, is how well a model can estimate the value of a dependent variable (DV) when the model does not already have access to that value. For example, the predictive accuracy of a time-preference test might signify how accurately the test can predict number of cigarettes smoked among people whose smoking has not been measured. Notice that the prediction in question is of individual data values (e.g., the number of cigarettes smoked by subject 3) in numeric terms (e.g., 7 cigarettes). Such predictions are distinct from what is usually meant when a researcher states that a theory “predicts” something: namely, ordinal effects such as “Less patient people smoke more.” Prediction, in the statistical sense of the term, need not be future-oriented: we can examine the predictive accuracy of one variable for another variable measured at the same time, or even before the predictor. What is important is not the timing of measurements, but that the model is not trained with the very same cases it is trying to predict.

This sort of criterion validity—predictive rather than merely associative—is especially useful in applied contexts, for assessment and decision-making. In such situations, tests are used for their ability to inform us about what we do not already know concerning the examinee. Test scores are used as tools to estimate these other, unknown quantities. Hence, the more accurate the test is in predicting the unknown quantities, the more useful it will be for guiding our decisions.

Predictive accuracy can be quantified with some of the same statistics as association, such as RMSE. However, association is optimistically biased as a measure of predictive accuracy, due to overfitting (Wasserman, 2004, Theorem 13.15; see also Arfer and Luhmann, 2015). Measuring predictive accuracy while avoiding this problem requires additional steps, such as cross-validation (Hastie et al., 2009, p. 228), which splits the data into parts for separate model-fitting and model evaluation. Without cross-validation or a similar technique, past studies on the criterion validity of time-preference tests provide little information as to how useful those tests would really be for predicting criterion variables (CVs) that have not yet been measured. The general problem of using a single dataset for both fitting a model (training) and evaluating its performance (testing) at the same time can be described as using the same data to train and test.

Many past studies have claimed to find that a test of time preferences, or at least a test of patience, can indeed predict CVs. These are only a subset of the large literature showing association between time preferences and CVs. But generally, they have not used appropriate methods to assess and quantify predictive accuracy, so they are not informative as to the question of prediction, despite the intentions of the authors. For example, Daugherty and Brase (2010) had college students complete a forced-choice patience measure (that of Kirby and Marakovic, 1996) and questions about health behavior, such as the use of tobacco and the frequency of dentist visits. They make the claim that patience (as well as other variables) predicts some of the health behaviors on the basis of (a) significant changes in *R*^2^ from hierarchical regression and (b) standardized regression coefficients that significantly differ from 0. But the *R*^2^ values were calculated using the same data to train and test. And regression coefficients are a distinct question from predictive accuracy: regression coefficients are only the best-fitting parameter values of a given model with a given fitting algorithm, and do not measure whether the model as a whole is predictively accurate, neither with those parameter values nor with different values. Finally, statistical significance is distinct from how accurate a model is at prediction. There are three ways *p*-values and the significance thereof fail to measure predictive accuracy. First, as discussed earlier, they do not separate training from testing. Second, *p*-values are not in units of the DV (e.g., number of cigarettes smoked). Third, even if predictive accuracy is held constant, $*p*$-values shrink as the sample size increases. These problems for the use of *p*-values for evaluating predictive accuracy are in addition to more general problems with *p*-values (e.g., Cohen, 1994; Cumming, 2014).

### 1.3. The present studies

The present studies sought to provide direct insight on the question of how to measure time preferences and how well tests of time preferences can predict CVs. Study 1 compared three families of time-preference tests on reliability, convergent validity, and ability to predict 10 self-reported CVs, ranging from overweight to credit-card debt. Subjects were 181 US residents recruited from the crowdsourcing website Amazon Mechanical Turk. Study 2 examined the predictive accuracy of another test of time preferences for 40 self-reported CVs, which covered many of the same content areas, as well as new areas such as flu vaccination and age of sexual debut. Subjects were thousands of people from the nationally representative National Longitudinal Survey of Youth 1979.

## 2. Study 1

To help answer the question of how best to measure time preferences, rather than manipulate a few administration details, we compared three representative families of time-preference tests. One family used the popular items of Kirby et al. (1999). This family is representative of the general practice of measuring time preferences with forced binary choices between SS and LL (Logan, 1965), and the particular items of Kirby et al. (1999) have appeared in many other studies since (e.g., Chabris et al., 2008; Kirby, 2009; Brody et al., 2014). Another family used an adaptive forced-choice procedure, the probabilistic bisection algorithm. This is a statistically sophisticated equivalent of stepwise procedures that attempt to home in on the subject's true preference (e.g., Mazur, 1987; Koffarnus and Bickel, 2014). The final family had subjects fill in a blank with an amount of money that would make them indifferent between two options. Such tests, which attempt to estimate points of indifference directly, are the most common kind of free-response econometric test (e.g., Kirby and Marakovic, 1995; Kimura et al., 2013). Importantly, each of the three families comprised two tests that allowed us to measure patience as well as nonstationarity.

We evaluated the preference tests on their retest reliability, their convergent validity, their association with a number of practically important CVs, and, crucially, their accuracy at predicting said CVs. In fact, we used predictive rather than associative methods for retest reliability and convergent validity as well as for criterion validity. We administered the preference tests three times, with the third administration separated from the first two by 1 month, allowing us to estimate retest reliability over immediate as well as 1-month intervals.

### 2.1. Method

The procedure was approved by the Stony Brook University Committee on Research Involving Human Subjects. All subjects provided informed consent. The committee waived the requirement for documentation of informed consent, since the study was conducted on Mechanical Turk.

#### 2.1.1. Preference tests

Subjects completed three families of tests of time preferences. Each family used a different approach to measure the same two theoretical constructs, patience (i.e., willingness to wait for larger rewards) and nonstationarity (i.e., the effect of front-end delays on willingness to wait). Each family comprised two very similar tests (for a total of six distinct tests), which differed only in whether a front-end delay of 1 month was added to all the options presented to subjects. Tests with this delay are termed the *far* tests, whereas tests without it are termed the *near* tests. In theory, the near and far tests alone each measure patience, whereas the difference between subjects' behavior in the near and far tests measures nonstationarity.

The first family, which we call the *fixed* tests, used the medium-magnitude items of Kirby et al. (1999). In 7 trials, subjects made a forced choice between an SS reward and an LL reward. The SS delays were always “today” in the near fixed test and “30 days” in the far fixed test. The LL delays were the same as in Kirby et al. for the near test but were incremented by 30 days for the far test. We scored each test similarly to Kirby et al. but with a less model-specific metric. Specifically, we represented each item by its rank in terms of discount rate of indifference (such that $54 vs. $55 was item 1, $47 vs. $50 was item 2, etc.) and computed for each subject the most consistent rank rather than the most consistent discount rate. We then doubled the rank scores and subtracted 2 to put the scores on a scale of 0 to 20 with all but a few subjects receiving integer scores.

We designed the second family, of *bisection* tests, to adaptively estimate each subject's 1-month discount factor; that is, the number *d* ∈ [0, 1] such that *dx* dollars delivered at one time is worth to the subject *x* dollars delivered 1 month later. Adaption was accomplished using the probabilistic bisection algorithm (Horstein, 1963; our implementation was based on Waeber, 2013, p. 14), which repeatedly queries at the posterior median (estimated in Bayesian fashion) of an unknown parameter. We set the tuning parameter *p*_*c*_, which represents the probability of the queried system giving the correct signal (i.e., the subject choosing the option that they on average prefer), to 3/4. In each of 20 trials, subjects chose between an LL reward randomly selected from $15, $16, …, $95, and an SS reward chosen to make a subject with the current estimate of the discount factor indifferent between SS and LL, rounded to the nearest dollar. Delays were “today” and “1 month” for the near test and “1 month” and “2 months” for the far test. The score for each test was the final value of the posterior median of the discount factor, on the original [0, 1] scale.

Finally, the family of *matching* tests used a free-response rather than a forced-choice format. In each of 10 trials, the SS reward amount was randomly selected from $1, $2, …, $95 and the subject was asked to “fill in the blank [viz., the LL amount] with an amount that makes the two options equally appealing to you; that is, an amount that makes you indifferent between the two options.” Subjects could enter amounts with up to 1-cent precision. As in the case of the bisection tests, delays were “today” and “1 month” for the near test and “1 month” and “2 months” for the far test. These tests were scored simply by taking the median of the SS amounts divided by the LL amounts, yielding again a discount factor on the scale of [0, 1].

Notice that all tests were scored in the direction with greater scores implying greater patience. Notice also that these scores, which we used for all analyses below, are not the same as model-specific measures common in the study of intertemporal choice such as the discount rate *k*^1^. The goal of this study was not to evaluate any one of the many models of intertemporal choice that have been proposed (Doyle, 2013); our focus was on tests rather than models. We have previously found evidence that a wide variety of models can successfully predict responses in a pure intertemporal-choice task (Arfer and Luhmann, 2015), but in that study, we did not try to predict other kinds of behavior, and prediction was within rather than between subjects.

To check that the selected numbers of trials for the bisection and matching families were adequate, we ran pilot studies in which we administered both tests in each family twice, to 14 users of Mechanical Turk per family. We examined the difference between the discount factors yielded by the first and second administration for each test. For the two families, 75 and 83% of differences, respectively, were less than 0.1 (on the scale of discount factors, from 0 to 1), which we judged to be adequate.

#### 2.1.2. Criterion questionnaire

Subjects self-reported about a wide variety of real-world behaviors that are in theory related to patience and self-control, as well as their demographics. Chabris et al. (2008) was our primary inspiration in choosing and writing questions. Questions were asked of subjects in the order shown here. The response format for each question is described after the question in square brackets.

- Are you male or female? [“Male” or “Female”]
- How old are you? [Integer]
- How tall are you? [Length, US or SI units]
- How much do you weigh? [Weight, US or SI units]
- Do you use tobacco? [“Yes” or “No”]
  ■ [Shown only if the subject answered “Yes”] How many packs of cigarettes do you smoke per week? (Enter 0 if you don't smoke cigarettes.) [Integer]
- How many hours per week are you physically active (for example, working out)? [Integer]
- For how many of your meals do you choose the amount or kind of food you eat with health or fitness concerns in mind? [Percentage]
- How many times per week do you use dental floss? [Integer]
- Have you used a credit card at all in the past 2 years? [“Yes” or “No”]
  ■ [Shown only if the subject answered “Yes”] Over the past 2 years, how many times were you charged a late fee for making a credit card payment after the deadline? [Integer]
  ■ [Shown only if the subject answered “Yes”] Over the past 2 years, how many of your credit-card payments were for less than your total balance? [Percentage]
- Over the past 2 years, how much of your income have you saved? (Please include savings into retirement plans and any other form of savings that you do.) [Percentage]
- On how many days per month do you gamble? (Gambling includes such activities as playing at casinos, playing cards for stakes, buying lottery tickets, and betting on sports.) [Integer]

The theoretical relationship of the CVs to time preferences, and thus self-control, is that each is related to choices between small rewards available soon and larger rewards available later. For example, smoking entails getting the immediate pleasure of a cigarette and forgoing the long-term health benefits of not smoking. Overweight is related to overeating, which again entails getting immediate pleasure and forgoing long-term health benefits. And credit-card debt is accumulated by making immediate purchases with the effect of having to pay much more than the original purchase price over the long term. No variable is supposed to be caused by self-control alone, but each involves self-control partly.

#### 2.1.3. Subjects

Subjects were recruited from the crowdsourcing website Amazon Mechanical Turk. They were required to live in the United States. Of the 200 subjects who participated in session 1, 74 (37%) were female, and the median age was 31 (95% sample interval 19–64). All 200 were invited to complete session 2, and 103 did so. Subjects provided informed consent before each session. They were compensated with $1 for completing session 1 (median completion time 15 min) and $0.50 for completing session 2 (median completion time 6 min).

#### 2.1.4. Procedure

Session 1 took place from February 15th to 21st, 2014. In session 1, subjects completed all six preference tests in a random order (round 1), then did so again in another random order (round 2), with no delay in between or notification that the same tests were applied twice. (The order of tests was randomized per subject.) Finally, subjects completed the criterion questionnaire.

Without prior warning, subjects were invited to participate in session 2 by email approximately 30 days later. To balance server load, invitations were sent out in 5 batches of 10 per day starting on March 18th, 2014, so all participants in session 1 had been invited back by March 22nd. Subjects had until March 25th to complete session 2. This session consisted entirely of a third administration of the six preference tests, again in random order (round 3).

As an extra check on subject attention, we included in each bisection test two catch trials. In one catch trial, the ratio of amounts (SS amount divided by LL amount) was set to 0.07, making LL clearly preferential for all but the most impatient of subjects. In the other catch trial, the ratio of amounts was set to 1.13, making one option have both the lesser delay and the greater amount, giving subjects with no clear justification to prefer the other option. Choices in these trials were not used for scoring any tests.

### 2.2. Results and discussion

The raw data for both Study 1 and Study 2, as well as task code and analysis code, can be found at http://arfer.net/projects/rickrack.

Of the 200 subjects who participated in session 1, we excluded from analysis 4 subjects who gave a nonsensical answer to a question in the criterion questionnaire, 10 subjects who took the designated incorrect choice in at least 3 of the 8 catch trials in session 1, and 6 subjects who gave an LL response smaller than SS in at least 3 of the 40 matching trials in session 1. (No subjects made 3 or more errors in either category in session 2.) Accounting for overlap in these groups, 181 subjects remained. These 181 subjects constitute the sample for the bulk of the following analyses.

Of the 103 subjects who participated in both sessions, 7 were already excluded by the above rules, and we excluded an additional 3 subjects who completed session 2 in less than 3 min, for a final sample size of 93. This smaller sample of 93 is used only for the 1-month retest reliability analysis (the right-hand half of **Table 3**), since this is the only analysis using data from round 3 (i.e., session 2)^2^.

Descriptive statistics for preference-test scores in round 1 are shown in Table 1. The median completion time for session 1 was 15 min, and the median completion time for session 2 was 6 min.

**Table 1:** **Descriptive statistics for preference-test scores in round 1**.

	**Matching**		**Fixed**		**Bisection**	
	**Near**	**Far**	**Near**	**Far**	**Near**	**Far**
Q3	11	11	0.88	0.87	0.87	0.84
Median	9	9	0.76	0.77	0.69	0.66
Q1	7	5	0.59	0.58	0.50	0.50
MAD	2	2	0.15	0.16	0.18	0.16

*Q3, top quartile; Q1, bottom quartile; MAD, median absolute deviation from the median*.

We transformed and coded the CVs as follows, based on inspection of their distributions (without reference to the preference tests), so as to retain variability while maximizing their suitability as a DV for linear regression, dichotomous probit regression, or ordinal probit regression.

- Hours of exercise per week was incremented by 1 and log-transformed.
- Healthy meals and savings were clipped to [0.005, 0.995] and logit-transformed.
- Overweight was calculated by computing body mass index (as weight in kilograms divided by the square of height in meters), then dichotomizing using a threshold of 25.
- Gambling, credit-card late fees, and credit-card subpayments were dichotomized according to whether the subject's answer was greater than 0.
- Number of cigarettes smoked was ignored in favor of the dichotomous variable of whether the subject used tobacco.
- Flossing was coded into three ordered categories: 0 (less than once per week), 1–6 (less than once per day), and 7 or more (once or more per day).

#### 2.2.1. Association with criterion variables

For each CV and family of preference tests, we assessed how well the preference tests (administered in round 1) could account for variation in the CV with a regression model. Each model had four terms: an intercept, main effects for the near and far test scores, and an interaction. The model was an ordinary linear regression model for the continuous CVs (exercise, healthy meals, and savings), a dichotomous probit regression model for the dichotomous CVs (overweight, tobacco, gambling, credit-card late fees, and credit-card subpayment), and an ordinal probit regression model for flossing. Models with a CV related to credit cards included only subjects who stated they had used a credit card in the past 2 years.

Measures of model fit are shown under “Association” in Table 2. These values of *R*^2^ and Efron's *R*^2^ are small, but similar to those obtained by Chabris et al. (2008). The bisection tests achieve the greatest fit for 7 of the 9 CVs.

**Table 2:** **Results for assocation and prediction between each family of preference test and criterion variable (CV)**.

	**Continuous**			**Discrete**					
	**Exercise**	**Healthy meals**	**Savings**	**Overweight**	**Tobacco use**	**Gambling**	**Flossing**	**CC late fees**	**CC subpayment**
**ASSOCIATION**									
Fixed	0.026	0.037	0.004	0.005	0.019	0.025	0.009	0.025	0.012
Bisection	0.084	0.054	0.025	0.002	0.035	0.055	0.004	0.033	0.013
Matching	0.035	0.040	0.013	0.001	0.003	0.040	0.009	0.015	0.038
Baseline	6.880	0.335	0.170	0.560	0.760	0.830	0.360	0.750	0.600
**PREDICTIVE ACCURACY**									
Fixed	7.110	0.348	0.188	0.490	0.750	0.820	0.350	0.730	0.570
Bisection	6.770	0.352	0.186	0.520	0.760	0.820	0.280	0.740	0.540
Matching	7.150	0.360	0.188	0.560	0.760	0.820	0.330	0.740	0.580

*Association is measured with R^2^ for continuous CVs and Efron's R^2^ for discrete CVs. The baseline measure is standard deviation for continuous CVs and the base rate of the modal class for discrete CVs. Predictive accuracy is measured with root mean squared error for continuous CVs and proportion agreement for discrete CVs. CC, credit card*.

#### 2.2.2. Prediction of criterion variables

In the previous section, we assessed the association of preference tests with CVs. Here, by contrast, we assess how accurately the tests could predict unseen data. To do this, we subjected the same models just described to tenfold cross-validation. The results are shown under “Predictive accuracy” in Table 2. Observe that none of the models performed above baseline. For the discrete CVs, none of the models had a higher proportion of correct predictions than the base rate of the modal class. For the continuous CVs, in only one case was the RMSE less than the standard deviation of the CV, and in that one case (of the bisection tests predicting exercise), the improvement was tiny. Thus, these preference tests appear to be of limited value for predicting these CVs.

We reasoned that if there really existed a predictively useful relationship between the preference tests and CVs, perhaps it was too complex to be exploited by these models. We thus tried several more complex procedures: *k*-nearest neighbors classification and support vector machines for the discrete CVs, locally linear kernel regression for the continuous CVs, and random forests for all CVs. Like the simpler regression models, these procedures at best barely improved upon baseline. We omit further details for space.

#### 2.2.3. Retest reliability

The notion of reliability most closely related to predictive criterion validity is a test's accuracy in predicting itself. We therefore assessed the retest reliability of our preference tests by assessing how well scores in round 1 predicted scores in rounds 2 and 3. We attempted to predict round-2 and round-3 scores with unaltered round-1 scores rather than using a statistical model, since the question is how stable the scores are on their own. By contrast, Pearson correlations, for example, do not penalize bias. A convenience of this approach is that cross-validation is not necessary for estimating predictive accuracy, because there is no model to train.

Our three rounds of preference testing in two sessions allowed for estimating retest reliability over two intervals, immediately and 1 month (Table 3). We calculated bias as the mean difference between the target score and the predictor score. In our judgment, these are moderate to high degrees of reliability. Subjects' scores did not change much either immediately or over the course of a month, with median absolute errors around 0.05 for the bisection and matching tests. Bias for all tests was low. As for between-test differences in reliability, the only clear difference these findings suggest is that the matching tests had greater immediate retest reliability than the other two families, and this may be an artifact of how it is easier to remember and reuse a response strategy for this family than for the other two. But also recall that in the fixed family, unlike the bisection and matching families, every administration of the same test presents the same items. This means that reliability estimates for the fixed family, particularly over the immediate interval, may be inflated by subjects remembering and reusing their past answers.

**Table 3:** **Accuracy of round-1 preference tests used to predict scores on the same tests in round 2 (immediately after round 1 in session 1) and round 3 (in session 2, a month after session 1)**.

	**Predicting round 2**						**Predicting round 3**					
	**Fixed**		**Bisection**		**Matching**		**Fixed**		**Bisection**		**Matching**	
	**Near**	**Far**	**Near**	**Far**	**Near**	**Far**	**Near**	**Far**	**Near**	**Far**	**Near**	**Far**
PVAF	0.70	0.49	0.51	0.62	0.75	0.82	0.44	0.57	0.44	0.64	0.57	0.56
Abs err, median	2.00	2.00	0.04	0.05	0.03	0.03	2.00	2.00	0.06	0.04	0.06	0.05
Abs err, 90th %ile	5.00	7.00	0.23	0.18	0.14	0.13	5.00	5.00	0.16	0.19	0.19	0.21
Abs err, 95th %ile	5.00	9.00	0.28	0.24	0.20	0.18	7.00	6.00	0.21	0.23	0.28	0.26
Bias	0.10	0.09	0.00	−0.01	−0.01	−0.01	−0.37	−0.29	0.01	0.01	−0.02	−0.02
Kendall τ	0.71	0.61	0.65	0.63	0.74	0.78	0.66	0.65	0.66	0.67	0.65	0.62
Pearson *r*	0.85	0.72	0.76	0.81	0.88	0.91	0.77	0.80	0.72	0.82	0.81	0.79

*PVAF, proportion of variance accounted for; Abs err, absolute error*.

Table 3 also includes Pearson correlations, in spite of the foregoing discussion, for the sake of comparability with past studies. These are around 0.8 for both intervals, which is similar to past studies of intertemporal-choice tests and compares favorably to personality tests in general (Schuerger et al., 1982).

#### 2.2.4. Convergent validity

If our tests could predict themselves but not external CVs, could they predict each other? We estimated convergent validity by examining mutual prediction in round 1 with tenfold cross-validated linear regression (clipping predictions to the legal range of the DV). We found moderate convergent validity, with proportions of variance accounted for (PVAF) ranging from 0.25 to 0.50. These figures are comparable to those of Smith and Hantula (2008), who found that free-response and forced-choice patience tests were correlated 0.33 (PVAF 0.11) in area under the curve and 0.75 (PVAF 0.56) in discount rate. Generally, however, these figures are substantially lower than the corresponding immediate retest reliabilities. Therefore, as would be expected, the tests can predict one another to some degree but also have individual components of variance. The different families are measuring similar but not identical constructs.

#### 2.2.5. Nonstationarity

Descriptively, what nonstationarity did subjects exhibit in their responses to the preference tests? Table 4 examines nonstationarity in round 1 by comparing subjects' near scores and far scores for each test. Greater scores (more patience) on the far test than the near test indicate classical nonstationarity, in which the agent plans to be more patient in the future than the present, whereas the reverse indicates the less theoretically appealing phenomenon of planning to be less patient in the future than the present, and equal scores on both tests indicate perfect stationarity. (Perfect stationarity necessarily appears more often for the fixed family than the other two because of its discrete scoring scheme.) As can be seen in the table, there was no overall trend in any of the three families for subjects to plan to be more patient later. If anything, subjects tended to plan to be less patient later. These findings are contrary to theory that requires decision-makers to be more patient in the far test, but consistent with past reports that have found no strong trend in either direction (Ahlbrecht and Weber, 1997; Read, 2001; Kable and Glimcher, 2010).

**Table 4:** **Counts of subjects exhibiting each kind of nonstationarity on each family of preference tests in round 1**.

	**Fixed**	**Bisection**	**Matching**
Less patient later	75	95	100
Stationary	64	0	10
More patient later	42	86	71

To examine whether the presence of the front-end delay could make a difference for prediction, we conducted an analysis similar to the reliability analyses described earlier with round-1 near scores as the predictor and round-2 far scores as the DV. The results (Table 5) are similar to the prediction of the same DV (round-2 far) with round-1 far (Table 3) rather than round-1 near, particularly in terms of Kendall τs. This implies that scores on near tests can predict scores on far tests roughly as well as they can predict themselves. This in turn suggests that there is no special predictive value in using the separate near and far tests; one might as well use twice as many trials of a near test and get the same predictive ability for any CV of interest.

**Table 5:** **Accuracy of round-1 near tests used to predict scores on round-2 far tests**.

	**Fixed**	**Bisection**	**Matching**
PVAF	0.47	0.42	0.68
Abs err, median	2.00	0.05	0.04
Abs err, 90th %ile	7.00	0.22	0.17
Abs err, 95th %ile	9.00	0.31	0.23
Bias	0.46	−0.02	−0.01
Kendall τ	0.58	0.60	0.73
Pearson *r*	0.70	0.75	0.86

*PVAF, proportion of variance accounted for; Abs err, absolute error*.

## 3. Study 2

In Study 1, none of our tests of time preferences could predict any of the CVs with more than trivial accuracy. The findings for retest reliability and convergent validity support the quality of the time-preference tests. For the CVs, however, we do not have this psychometric information, raising the possibility that the low predictive accuracy resulted from something unusual or deficient about the 10 items we happened to use, which were based heavily on the items of Chabris et al. (2008). In Study 2, we analyzed a large public dataset with a much larger number and variety of CVs. The data of Study 2 has the additional benefit of using in-person interviews rather than Internet questionnaires, perhaps motivating subjects to answer questions more seriously and thoughtfully.

### 3.1. Method

The National Longitudinal Survey of Youth 1979 (NLSY79; http://nlsinfo.org) is a project by the US Bureau of Labor Statistics, in which people in a nationally representative sample of Americans born between 1957 and 1964 are periodically interviewed. Questions asked of subjects have differed over the 25 interview rounds. The 2006 interview (and no others for which data is yet available), at which time subjects were ages 41–50, included two questions intended to measure time preferences, particularly patience: IMPATIENCE_1 (reference number T09617.00) and IMPATIENCE_2 (reference number T09620.00). The first of these asked:

Suppose you have won a prize of $1000, which you can claim immediately. However, you can choose to wait 1 month to claim the prize. If you do wait, you will receive more than $1000. What is the smallest amount of money in addition to the $1000 you would have to receive 1 month from now to convince you to wait rather than claim the prize now?

The second item was the same except with an interval of 1 year instead of 1 month. Subjects could answer with any nonnegative integer. In theory, greater responses indicate less patience, and as in Study 1, a comparison of responses between the two timepoints should capture nonstationarity. Of 7,649 subjects interviewed, 7,127 provided a valid response to both questions. The remainder, who refused to answer one of the two questions or said they did not know, were given follow-up questions asking them to provide a range estimate. It was not clear to us how to use range estimates alongside the usual responses, and relatively few subjects (less than 30 for each of the month and year scenarios) provided a range estimate, so we restricted our analyses to the 7,127 subjects who answered the month and year questions with integers. To remain neutral on the question of how to model intertemporal choice itself (as in Study 1), we used the responses directly rather than fitting them to a model of intertemporal choice such as hyperbolic discounting.

Of these 7,127 subjects, 51% were female. Regarding race, 52% were white, 30% were black, 19% were Hispanic, and 1% were Asian (subjects could endorse more than one category). The median net family income (which we also use as a CV) was $54,975. Among the 93% of subjects who the surveyors could determine as living in a rural area or an urban area in 2006, 29% lived in a rural area.

For CVs, we searched the NLSY79 for items concerning real-world self-control, covering both the domains considered in Study 1 (obesity, exercise, drug use, healthy eating, oral hygiene, debt, and saving; we found no items concerning overall gambling behavior) and new domains (sleep, health insurance, vaccination, sexual debut, divorce, crime, and income). This search produced a list of 1,034 items, some of which were the same question asked in different years or were otherwise indistinct from other items, and some of which were not asked of any subjects who answered the patience items. Below, we describe the 40 variables we produced from these items. When items were available for multiple years, we preferred years closer to 2006 (the year the patience questions were asked), breaking ties in favor of years after rather than before 2006.

### 3.2. Results and discussion

As in Study 1, we transformed and coded the CVs based on inspection of their distributions (without reference to the preference tests) so as to retain variability while maximizing their suitability as DVs. Table 6 reviews the 40 CVs we produced; see the NLSY79 documentation for details of the original survey questions. In our analyses, we included only cases that were missing on neither the predictors nor the CV. The resulting sample sizes were still large, ranging from 3,100 to 7,127 (median 6,767).

**Table 6:** **The criterion variables used in Study 2**.

	***n***	**Year**	**Scale**	**Description**
Difficult to run mile	4,932	XRND	Binary	S said they could not or did not run a mile, or that it was very difficult
Not easy to climb stairs	4,995	XRND	Binary	S rated climbing stairs as more difficult than “Not at all difficult”
Overweight	6,923	2006	Binary	Body mass index 25 or more
Exercise, light, ever	6,789	2006	Binary	S reported nonzero frequency of light or moderate exercise
Exercise, light, min/y	5,012	2006	Continuous (log)	Calculated minutes per year of light or moderate exercise (nonzero only)
Exercise, vigorous, ever	6,855	2006	Binary	S reported nonzero frequency of vigorous exercise
Exercise, vigorous, min/y	4,667	2006	Continuous (log)	Calculated minutes per year of vigorous exercise (nonzero only)
Exercise, strength, ever	7,097	2006	Binary	S reported nonzero frequency of strength training
Checks nutrition often	7,053	2006	Binary	S “often” or “always” reads nutritional info while shopping
Eats fast food	6,858	2008	Binary	S ate fast food at least once in past week
Drinks soft drinks	6,850	2008	Binary	S drank a (non-diet) soft drink at least once in past week
Sleep min, weekday	4,997	XRND	Continuous	Minutes of sleep S usually gets on weekdays
Sleep min, weekend	4,994	XRND	Continuous	Minutes of sleep S usually gets on weekends
Health insurance	7,123	2006	Binary	S has health insurance
Flu vaccine	6,852	1979	Binary	S received flu vaccine in past 2 years
Sees dentist	6,856	1979	Binary	S saw a dentist in past 2 years
Brushes teeth 2/day	6,551	2008	Binary	S brushes teeth twice daily
Flosses daily	6,544	2008	Binary	S flosses daily
Smoked 100 cigs	6,855	2008	Binary	S smoked 100 cigarettes in lifetime
Smoking	6,856	2008	Binary	S smokes “occasionally” or “daily”
Drinking	7,052	2006	Binary	S drank alcohol in past month
Drinking, heavy	7,045	2006	Binary	S drank more than 6 drinks in one occasion in past month
Drinks in last month	3,691	2006	Continuous (log)	Calculated number of drinks in past month (nonzero only)
Cannabis	6,662	1998	Binary	S ever used cannabis
Cocaine	6,690	1998	Binary	S ever used cocaine
Stimulants	6,711	1998	Binary	S ever used stimulants recreationally
Other drugs	6,694	1980	Binary	S ever used illegal drugs (other than cannabis)
Sexual debut	6,563	1979	Continuous	Age S first had “sexual intercourse”
Divorced	7,127	XRND	Binary	S ever divorced or separated
Stopped by police	6,907	1980	Binary	S ever stopped by police (other than for minor traffic violation)
Convicted	6,910	1980	Binary	S ever convicted (other than for minor traffic violation)
Net family income	6,748	2006	Continuous (sqrt)	Calculated net family income in previous calendar year (top-coded)
Saving	6,618	2000	Binary	S or partner has money in bank account or US savings bonds
Retirement account	6,599	2000	Binary	S or partner has money in IRA, Keogh, 401(k), etc.
Missed bill payment	6,831	2008	Binary	S missed or was 2 months late to a bill in past 5 years
CC debt, any	6,609	2008	Binary	S or partner had nonzero CC balance after most recent payment
CC debt, dollars	3,100	2008	Continuous (log)	Dollars of credit-card debt (nonzero only)
CC maxed out	6,775	2008	Binary	S or partner has a maxed-out credit card
Debt to businesses	6,811	2008	Binary	S or partner in debt to a store, hospital, bank, etc.
Negative net worth	6,759	2008	Binary	S's liabilities exceed S's assets

*“n” is the number of non-missing cases. “Year” shows the survey year in which the corresponding questions were asked; “XRND” means that questions from multiple survey years were used (some health questions were only asked of subjects once they reached age 50, so we used the available assessment for each subject whichever year it was asked on, and our divorce item is inferred from assessments of marital status that were made repeatedly from 1979 to 2012). “Scale” indicates whether the variable is binary or continuous, and if it is continuous, what transformation, if any, was applied before fitting models. S, subject; CC, credit card*.

To ensure we replicated past studies' findings of significant association between CVs and patience, we ran Wilcoxon rank-sum tests for the binary CVs (treating patience as the DV rather than as the predictor, as is usual in this literature) and Kendall correlation tests for the continuous CVs. We chose nonparametric tests so any monotonic relationship could be detected, regardless of scaling. Two tests were run for each CV, one for month patience and one for year patience. We found that of the 64 tests for the binary CVs, 47 were significant (34 after a Holm-Bonferroni correction was applied for 64 comparisons), and of the 16 tests for the continuous CVs, 5 were significant (all of which remained significant after a Holm-Bonferroni correction for 16 comparisons). Exactly which tests were significant is indicated by superscripts in Tables 7, 8. What is important is that, as in past studies, we were able to find many significant associations between CVs and time preferences.

**Table 7:** **Results for binary criterion variables in Study 2**.

	**Base rate**	**Log model**			**Nominal model**		
		**Efron's *R*^2^**	**PA, a**.	**PA, p**.	**Efron's *R*^2^**	**PA, a**.	**PA, p**.
Difficult to run mile^m^^,^ ^y^	0.524	0.005	0.538	0.527	0.064	0.574	0.491
Not easy to climb stairs^mm^^,^ ^y^	0.604	0.008	0.603	0.602	0.071	0.629	0.581
Overweight^mm^^,^ ^yy^	0.717	0.004	0.717	0.716	0.048	0.723	0.705
Exercise, light, ever^mm^	0.738	0.013	0.738	0.738	0.073	0.746	0.729
Exercise, vigorous, ever^mm^^,^ ^y^	0.681	0.011	0.682	0.682	0.069	0.692	0.668
Exercise, strength, ever^mm^^,^ ^y^	0.628	0.009	0.627	0.625	0.062	0.646	0.607
Checks nutrition often^mm^^,^ ^yy^	0.514	0.010	0.539	0.539	0.060	0.579	0.524
Eats fast food^mm^^,^ ^y^	0.646	0.004	0.646	0.646	0.054	0.660	0.632
Drinks soft drinks^mm^^,^ ^yy^	0.579	0.010	0.580	0.579	0.065	0.613	0.577
Health insurance^mm^^,^ ^yy^	0.810	0.020	0.810	0.810	0.075	0.814	0.804
Flu vaccine^m^^,^ ^y^	0.681	0.003	0.681	0.681	0.052	0.691	0.662
Sees dentist^mm^^,^ ^yy^	0.668	0.021	0.670	0.668	0.073	0.681	0.652
Brushes teeth 2/day	0.741	0.001	0.741	0.740	0.058	0.748	0.732
Flosses daily	0.599	0.001	0.599	0.599	0.046	0.616	0.569
Smoked 100 cigs^m^^,^ ^y^	0.575	0.006	0.576	0.574	0.060	0.605	0.554
Smoking^mm^^,^ ^yy^	0.728	0.009	0.727	0.727	0.064	0.736	0.717
Drinking^mm^^,^ ^yy^	0.527	0.009	0.541	0.538	0.070	0.594	0.545
Drinking, heavy	0.859	0.000	0.859	0.859	0.051	0.860	0.850
Cannabis^mm^	0.617	0.004	0.617	0.616	0.055	0.638	0.605
Cocaine	0.767	0.003	0.767	0.767	0.052	0.771	0.749
Stimulants^mm^	0.887	0.005	0.887	0.887	0.058	0.889	0.882
Other drugs^m^	0.824	0.003	0.824	0.824	0.050	0.825	0.806
Divorced^mm^^,^ ^yy^	0.536	0.004	0.537	0.538	0.058	0.580	0.525
Stopped by police	0.824	0.003	0.824	0.824	0.058	0.826	0.810
Convicted	0.951	0.002	0.951	0.951	0.050	0.951	0.947
Saving^mm^^,^ ^yy^	0.716	0.034	0.715	0.714	0.097	0.729	0.705
Retirement account^mm^^,^ ^yy^	0.523	0.034	0.572	0.570	0.092	0.610	0.559
Missed bill payment^mm^^,^ ^yy^	0.787	0.010	0.787	0.787	0.062	0.791	0.769
CC debt, any^m^	0.531	0.005	0.541	0.537	0.059	0.587	0.533
CC maxed out^mm^^,^ ^yy^	0.887	0.006	0.887	0.887	0.062	0.889	0.881
Debt to businesses^m^^,^ ^yy^	0.796	0.005	0.796	0.796	0.058	0.799	0.779
Negative net worth^mm^^,^ ^yy^	0.885	0.014	0.885	0.885	0.065	0.886	0.877

*“Base rate” gives the base rate of the modal class. Efron's R^2^ is included for consistency with Study 1. PA, proportion agreement; a., association; p., prediction*.

m, mm, y, yy *Levels are associated with significantly different month or year patience at the 0.05 level (m and y, uncorrected; mm and yy, Holm-Bonferroni-corrected)*.

**Table 8:** **Results for continuous criterion variables in Study 2**.

	**MAD**	**Log model**		**Nominal model**	
		**MAE, a**.	**MAE, p**.	**MAE, a**.	**MAE, p**.
Exercise, light, min/y	25,940.94	25,921.10	25,940.86	25,427.31	26,886.97
Exercise, vigorous, min/y	22,205.79	22,173.85	22,197.58	21,730.30	23,316.54
Sleep min, weekday	63.32	63.18	63.46	60.37	66.33
Sleep min, weekend	73.66	73.18	73.34	69.09	76.23
Drinks in last month	17.92	17.91	17.95	17.34	18.80
Sexual debut^mm^^,^ ^yy^	1.91	1.91	1.92	1.83	1.99
Net family income^mm^^,^ ^yy^	44,145.32	43,151.85	43,285.46	40,976.40	44,268.49
CC debt, dollars^mm^	5,972.24	5,961.44	5,979.99	5,715.25	6,269.98

*For ease of interpretation, criterion variables have been transformed back to their original scales. R^2^, being specific to squared deviation whereas this analysis is in terms of absolute deviation, is not shown. MAD, mean absolute deviation from the median; MAE, mean absolute error; a., association; p., prediction*.

mm, yy *Kendall correlation with month or year patience is significant at the 0.05 level (Holm-Bonferroni-corrected)*.

As in Study 1, we examined the strength of association of time preferences with each CV and also the accuracy with which time preferences could predict CVs. Table 7 shows our results for the 32 binary CVs. We considered two models. The log model was a probit-regression model with 9 terms: an intercept, main effects for the logarithms of responses to the month and year patience items, dummy variables indicating responses of 0 to the month and year patience items, and all nontrivial one-way interactions. The nominal model treated every distinct pair of responses to the patience items as a class (i.e., one level of a nominal variable), with the exception that all singleton response pairs (response pairs that only one subject made) were unified into one class. There were 746 unique response pairs among the 7,127 subjects, of which 392 were singletons, for a total of 355 classes. The nominal model then predicted the CV by using whatever value of the CV was most common for that class in the training data, breaking ties by choosing the most common value in all the training data. The nominal model is thus essentially the most flexible model possible using these predictor variables. (We also examined two other models, a 3-term model with untransformed patience items and random forests. These generally performed worse than the log model, and are omitted for space).

As can be seen in Table 7, for the log model, both association and predictive accuracy were small. The association beats the base rate by 5 percentile points for having a retirement account (57 vs. 52%), and by 3 percentile points for checking nutrition often (54 vs. 51%); the remaining differences are below 2 percentile points. In fact, for six CVs, the association does not quite attain the base rate. Predictive accuracy is similar for having a retirement account (57%) and checking nutrition often (54%), but otherwise does not exceed the base rate by as much as 1 percentile point, and goes below the base rate for 16 CVs. Association is somewhat better for the more flexible nominal model, reaching a height of 61% agreement for having a retirement account. Predictive accuracy, however, is worse than that of the log model for all but two CVs, and the improvements for these two are each less than 1 percentile point. In short, the nominal model seems to overfit.

Table 8 shows our results for the 8 continuous CVs. We have performed the analysis in terms of absolute error rather than squared error so that extreme values of the CVs, of which there are several, are not weighted heavily. Thus, our baseline statistic is mean absolute deviation from the median (MAD) rather than SD; the log model is a quantile regression model predicting the CV with conditional medians, rather than a least-squares regression model predicting the CV with conditional means; and the nominal model uses medians rather than means of the values in its training set.

As can be seen in Table 8, the log model's strength of association always exceeds the MAD, but the improvement is very small (e.g., there is a difference of 29 s of weekend sleep). Proportional to the MAD, the largest difference is for net family income, $993, but this is still not much predictive accuracy given a MAD of $44,000. On the predictive side, the log model fails to beat the MAD for most of the CVs (5 of 8). The best MAD-proportional improvement is again for income, this time a slightly smaller $881. The nominal model achieves better association than the log model (including a $3,168 improvement from the MAD for income), but this again seems to be due to overfitting: the nominal model's predictive accuracy is inferior to the log model's for all CVs.

The overall picture is similar to that of Study 1: the available measures of time preferences cannot predict the available CVs with more than trivial accuracy (except, perhaps, in the case of retirement savings). Weak association under the log models is no doubt related to this; however, our findings for the nominal models exemplify the fact that stronger association is not sufficient for predictive accuracy.

## 4. General discussion

We assessed how accurately several tests of time preferences, comprising both patience and nonstationarity, could predict a variety of CVs. Study 1, using 181 users of Mechanical Turk, 10 CVs, and 3 distinct families of time-preference tests, found low to zero predictive accuracy. Study 2 replicated this finding for 7,127 participants in the NLSY79, 40 new CVs (covering all but one of the content areas of Study 1 as well as some others), and one new test of time preferences. The studies complement each other in that Study 1 took special care to ensure the quality of measurement of time preferences, whereas Study 2 used a much larger, nationally representative sample, and considered a richer set of CVs. In Study 1, we found that the three families of time-preference tests had decent retest reliability and convergent validity, supporting the idea that our negative result was not due to poorly chosen time-preference tests. In Study 2, we found that many of the relationships between time preferences and the CVs were significantly nonzero, exemplifying that significance does not imply predictive accuracy, and we found that the nominal model's greater strength of association did not translate into greater predictive accuracy, exemplifying the gap between association and prediction.

The consistently observed lack of predictive accuracy may be surprising in light of previous studies. However, as discussed in the introduction, past studies of time preferences have generally failed to evaluate predictive accuracy, despite the intentions of researchers. For, while these and other studies have found many significant associations between time preferences and CVs, significance and strength of association are distinct from predictive accuracy, as discussed in the introduction and demonstrated in Study 2. Hence, our findings are in no way *inconsistent* with these many previous findings. The difference, rather, is that we have specifically examined what many past studies might be thought to have examined—predictive accuracy—but did not.

Our finding of no predictive accuracy, which supports and strengthens the negative claim of Chabris et al. (2008), is important for the future study of real-world self-control behavior. It suggests that, regardless of the explanatory or descriptive merits of intertemporal-choice theory, time-preference tests are not useful—at least all on their own—for predicting such behavior. Researchers who wish to predict overeating, drug use, and debt accumulation should seek other variables to augment, if not replace, time preferences.

What can be made of Study 1's findings concerning reliability and convergent validity? They are useful as reassurance that we did not choose particularly poor tests, to which subjects responded mostly randomly. Arfer and Luhmann (2015) likewise showed that laboratory tests of time preferences can predict each other very accurately. It follows that the lack of predictive accuracy for CVs cannot be attributed solely to noise in the measurement of preferences or temporal instability in preferences.

Since nonstationarity plays an important role in economic thinking on self-control, but has been somewhat neglected in behavioral research on intertemporal choice, we took special care to include it in our tests. Not only did this not suffice for predictive accuracy for CVs, we found that preference tests could predict variations of themselves with different front-end delays just as well as they could predict themselves unaltered. This finding suggests there is little value in measuring stationarity separately from patience, supporting the usual practice of measuring only patience.

### 4.1. Limitations and future directions

Our conclusions are qualified by the limitations of our methods and the scope of our study. First, in terms of independent variables, we concerned ourselves exclusively with abstract time preferences. Even if time preferences are not predictively useful on their own, perhaps they have predictively useful interactions with other variables. Unfortunately, because there is no limit to the number and diversity of other independent variables that might be considered, there is no real way to falsify this idea. Another avenue we did not explore is measures of intertemporal choice that consider a domain other than money or that match the context of test-taking to the context of the behavior of interest. What is perhaps the most famous patience test uses marshmallows rather than money (Mischel et al., 1989). It is known that patience tests in which the reward is health (Chapman, 1996), sex (Johnson and Bruner, 2012), or percentage of a job offer spent on interesting tasks (Schoenfelder and Hantula, 2003) tend not to be strongly related to patience for money. And pathological gamblers are less patient when tested in an off-track betting parlor than when tested in neutral settings (Dixon et al., 2006). Perhaps domain- or context-specific tests of intertemporal choice will prove more predictively useful than generic time-preference tests. We may be able to predict obesity more accurately by asking for intertemporal choices about cookies in a kitchen than about money in a laboratory. This said, the bulk of existing research on intertemporal choice and its relation to criterion variables has, like our study, considered only intertemporal choice for money. So to the degree that this limitation applies to our own study, it applies to most of the existing literature as well.

All our measures of time preferences had subjects give judgments about hypothetical rather than real scenarios, which may seem questionable. However, past research contrasting real and hypothetical rewards has found no effect on time preferences (e.g., Johnson and Bickel, 2002; Madden et al., 2003, 2004; Lagorio and Madden, 2005). In a similar vein, our CVs were self-reported, which was consistent with many past studies. Self-report allowed us to include 50 different CVs across the two studies, but their reliability and accuracy may have been impaired by phenomena such as self-presentation concerns and subjects' imperfect memory for how often they exercise. We can at least say that unlike many self-report measures in psychological research, our CVs asked about objective phenomena (such as minutes of sleep) rather than requiring explicitly subjective judgments (such as how happy one feels), reducing the variability in how subjects could interpret and answer our questions.

Finally, in Study 1, we cannot know 1-month retest reliabilities among the many subjects who did not return for session 2. Our return rate, at 52%, was less than the 60% obtained in another retest-reliability study on Mechanical Turk by Buhrmester et al. (2011), but greater than the 28% obtained in the in-person retest-reliability study of Meier and Sprenger (2015).

## Author contributions

Both authors provided ideas, planned the study, and edited the manuscript. KA wrote task code and collected the data for Study 1, conducted analyses, and wrote initial drafts of the manuscript.

## Funding

This study was funded by the National Institute of Mental Health (T32MH109205) and the UCLA Center for HIV Identification, Prevention and Treatment Services (P30MH58107).

### Conflict of interest statement

The authors declare that the research was conducted in the absence of any commercial or financial relationships that could be construed as a potential conflict of interest.

## Footnotes

^1^We also generated Tables 2, 3 with each test represented not by its raw score, as described in this section, but by the natural logarithm of a discounting-rate parameter *k* obtained by fitting the data from each test administration to a model of hyperbolic discounting. Accuracy for predicting CVs was substantially similar. Immediate retest reliability was similar for the fixed and matching tests, but substantively reduced for the bisection tests.

^2^We regenerated all the tables for Study 1 in this paper using all 200 subjects (i.e., without any exclusion criteria, except of course that we could not assess 1-month retest reliability among subjects who did not return for session 2). We also regenerated Tables 2, 3 with stricter exclusion criteria, in which subjects who fell for even one catch trial or gave even one LL response smaller than SS were excluded, yielding a sample size of 137. All these results were substantially similar to those reported in the tables shown here.
